# Revisiting the Conundrum: A Case Report on Trauma Whipple's Pancreaticoduodenectomy

**DOI:** 10.7759/cureus.27189

**Published:** 2022-07-23

**Authors:** Adithya V Naragund, Rohith Muddasetty, Sharath S Kumar

**Affiliations:** 1 Surgical Gastroenterology and Hepatobiliary Surgery, Prakriya Hospitals, Bengaluru, IND

**Keywords:** american association for the surgery of trauma grading, trauma, aast grading, whipple’s pancreaticoduodenectomy, pancreatic ascites, traumatic pancreatitis, pancreatic trauma

## Abstract

Despite its rarity, pancreatic trauma is a serious condition because of its retroperitoneal location, association with other organ injuries, and complex bilio-vascular anatomy. Even less common are isolated pancreatic injuries. In grade four injuries, there is a debate over resectional vs. non-resectional management and appropriate treatment is particularly difficult. Here we discuss a patient with grade four pancreatic injury with pancreatic ascites presenting four days after the incident and traumatic pancreatitis. She underwent pylorus-preserving pancreatoduodenectomy and recovered well with acceptable morbidity.

## Introduction

The pancreas is an uncommon organ involved in abdominal trauma. Incidence is around 0.2% in blunt trauma and 1.1% in penetrating abdominal injuries [1]. Pancreatic injuries have a mortality rate of 12% and morbidity of 50%. The grade of pancreatic injury is the factor that determines morbidity and mortality [2]. The presence of pancreatic duct injury is a significant predictor of complications and prognosis [3]. The American Association for the Surgery of Trauma (AAST) has classified pancreatic trauma into five grades based on increasing severity as follows [4]: (i) Grade one, minor contusion without ductal injury and/or superficial laceration without ductal injury, (ii) Grade two, major contusion without ductal injury or tissue loss and/or major laceration without ductal injury or tissue loss, (iii) Grade three, distal transection or pancreatic parenchymal injury with ductal injury, (iv) Grade four, proximal transection or pancreatic parenchymal injury involving the ampulla, and (v) Grade five, massive disruption of the pancreatic head.

Several articles have been published on the treatment of pancreatic injuries, broadly summarized as non-operative management of grade one or two injuries and operative management for other grades [5]. However, resection in the setting of delayed presentation or after the onset of pancreatitis is controversial. Here, we report a case in which the patient presented four days after the injury when acute pancreatitis had already set in.

## Case presentation

**Figure 1:**
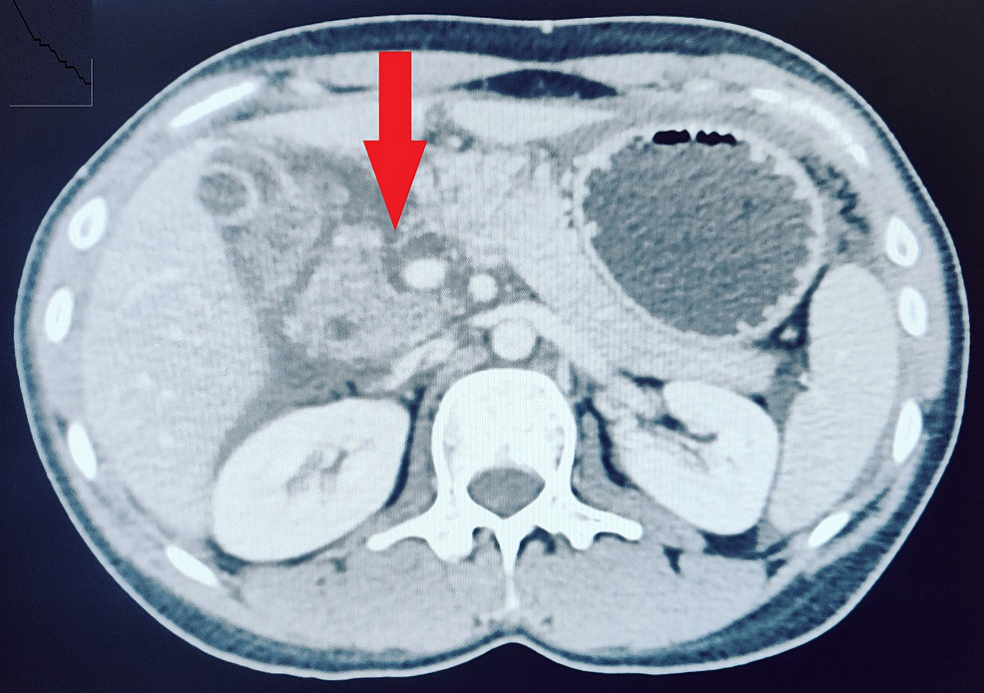
CT image showing pancreatic injury

A 29-year-old female presented to the emergency room with a history of assault over the abdomen four days ago. On presentation, she was tachycardic and had severe pain in the abdomen. Examination revealed guarding and distension of the abdomen. There were no external signs of bruising on the abdomen. The patient had visited multiple hospitals over the past four days and had been diagnosed with a pancreatic neck transection (Grade 4) on CT scan of abdomen done two days ago (Figure 1). There was no radiological evidence of associated injuries. Initial CT had shown mild ascites. She was admitted and managed conservatively in other hospitals and referred to our center once the pain in the abdomen and ascites increased.

**Figure 2:**
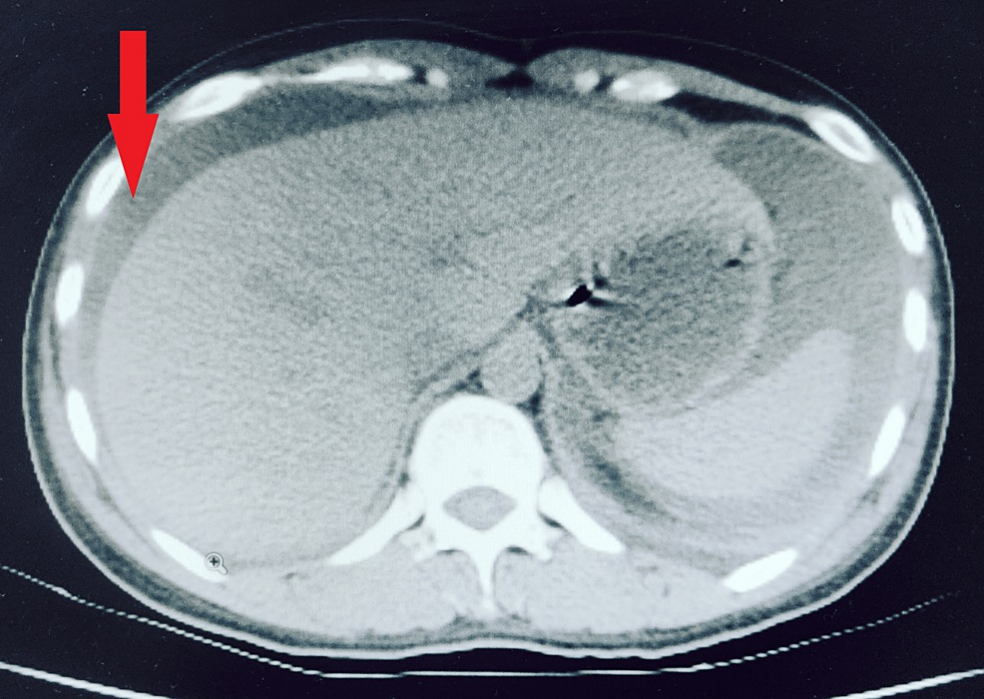
CT image showing ascites marked by arrow

**Figure 3:**
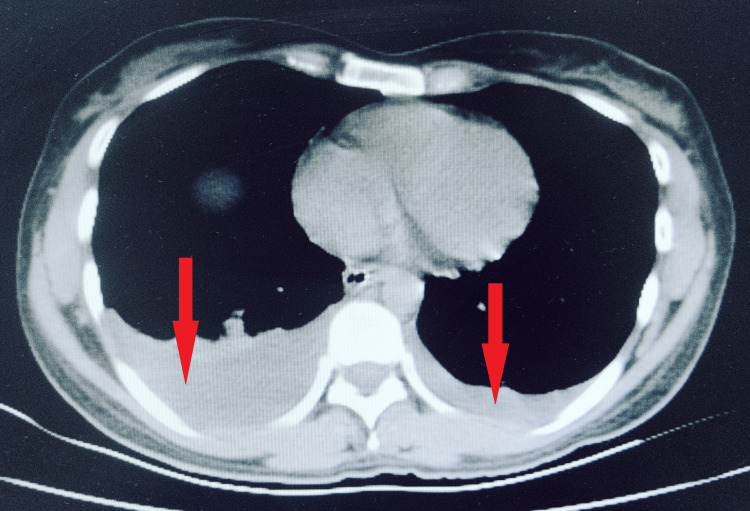
CT image showing bilateral pleural effusion marked by arrows

After initial stabilization, a plain CT of the abdomen was done, which showed massive ascites with bilateral moderate pleural effusion (Figures 2, 3). Ascitic fluid analysis revealed very high amylase levels of 5000U/L. Blood examination also revealed high serum amylase of 650U/L and lipase of 2478U/L (Lipase > Amylase) suggestive of acute pancreatitis. Her C-reactive protein at admission was 265U/L. The presence of local complication in form of ascites and the absence of organ failure suggested moderately severe pancreatitis. Other blood parameters were essentially normal except for hypocalcemia (secondary to pancreatitis).

The patient was started on antibiotics and octreotide infusion. After initial resuscitation for 24 hours, the tachycardia settled down. After careful consideration and review of the scan, in view of complete pancreatic neck transection and the guarding and rigidity, a decision to explore was taken. The therapeutic options considered were (i) drainage only, (ii) staged resection (stage1) and reconstruction (stage 2), (iii) upfront complete Whipple's procedure, or (iv) distal pancreatectomy.

**Figure 4:**
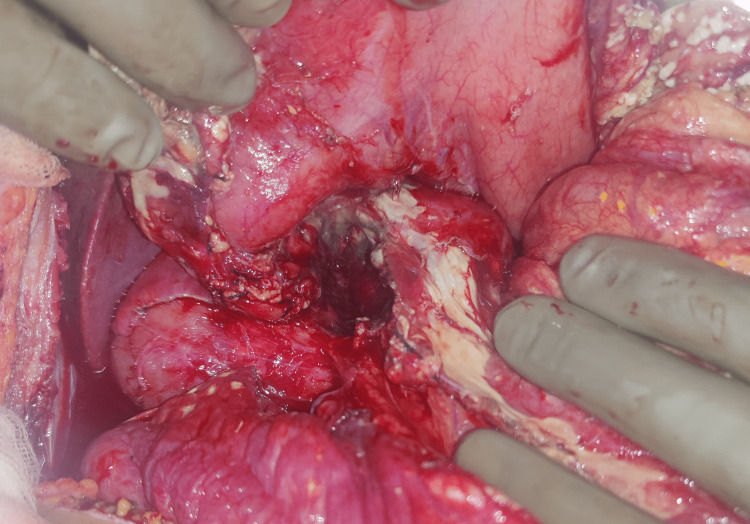
Intraoperative image showing saponification, pancreatic transection, and underlying portal vein

Intraoperatively, we found approximately eight litres of clear straw-coloured fluid, which was suctioned out, with no hemoperitoneum. There was evidence of ongoing acute pancreatitis with saponification of the omental fat (Figure 4) and extensive adhesions in the lesser sac. The neck of the pancreas was completely transected exposing an intact superior mesenteric vein and portal vein (Figure 4). The head of the pancreas was necrotic and there was some inflammation seen in the transected area. In view of the patient being hemodynamically stable, the morbidity, and the cost of two-staged procedures, the decision was taken to perform pylorus-preserving pancreatoduodenectomy.

The pancreatic neck margin was revised and a duct of one millimetre in size was identified. However, due to active inflammation, sutures would not hold and, hence. a dunking pancreaticojejunostomy was performed using polydioxanone (PDS 3.0, Ethicon, Inc., Raritan, New Jersey, United States) and silk sutures. No stent was placed. Hepaticojejunostomy was performed in a standard continuous way and duodenojejunostomy and Braun jejunojejunostomy were performed. A nasojejunal tube was placed as a standard practice to facilitate feeding. Two wide bore drains, 28Fr and 32Fr, were placed in the Morrison’s pouch and anterior to the pancreatojejunostomy respectively. In addition, we placed a 16Fr drain adjacent to these two drains for continuous irrigation in the post-operative period with the hope of diluting the pancreatic fluid in case of a leak and to keep the drains patent. Risk factors for a pancreatic leak in this patient were small duct, soft pancreas, active pancreatitis, and dunking technique of anastomosis.

The postoperative period was uneventful. Irrigation and octreotide were continued for five days. The patient was mobilized on the first postoperative day and started on nasojejunal feeds on day two. Day five drain fluid amylase was 2595U/L, which was followed by a CT scan of the abdomen showing no intra-abdominal collection suggestive of biochemical leak. The patient was discharged on day eight with one drain in situ. During follow-up, the drain was removed on postoperative day 14. She developed a surgical site infection, which was managed with regular dressing. At the one-month follow-up, the patient was healthy, able to tolerate normal diet, the wound has completely healed, and the screening ultrasound abdomen showed no intra-abdominal collection.

## Discussion

Abdominal trauma is one of the common causes to present in the emergency room. Being located in the retroperitoneum, the pancreas is an uncommon organ to be involved in blunt trauma. Isolated pancreatic injuries are also uncommon. Presentation can be in the form of incidental findings on laparotomy done for hemodynamic instability, can be found on imaging done for a hemodynamically stable patient, or may be a delayed presentation as acute pancreatitis, fluid collections, ascites, or pseudocyst. Initial management follows the trauma protocol of maintaining an adequate airway, breathing, and circulation. Definitive management depends on the grade of pancreatic injury and the presence of associated other organ injuries.

Phillips et al. (2016) have formulated guidelines for the management of pancreatic injuries based on the grade and type of presentation (Eastern Association for the Surgery of Trauma (EAST) guidelines), which have been summarized as follows: Grades one or two pancreatic injuries identified on imaging would require only conservative management. If these injuries were to be identified intraoperatively, non-resectional management is advised. Grades three or four pancreatic injuries either identified on imaging or intraoperatively should be subjected to resection procedures as the morbidity in form of pancreatic fistula, ascites, or treatment failure is higher in non-resectional management. Literature on the management of Grade five pancreatic injuries is sparse, thus formulation of guidelines would be difficult owing to high mortality [5].

Conservative management of pancreatic injuries has been advocated by many authors who argue against operative management. Abdo et al. have described the SEALANTS (Somatostatin, External drainage, ALternative nutrition, Antacids, Nil-per-os, Total parenteral nutrition, and a Stent in the pancreatic duct) approach to managing pancreatic duct disruption. They claimed that seven out of 12 patients had recovered within 40 days. However, patients in this were not restricted to trauma alone [6]. Studies have been summarised in Table 1. 

**Table 1:** Table describing the findings and conclusions of various studies in the literature SEALANTS: somatostatin, external drainage, alternative nutrition, antacids, nil-per-os, total parenteral nutrition, and a stent in the pancreatic duct

S. No.	Studies on pancreatic trauma	Conclusion
1.	Abdo et al. [6]	Seven out of 12 patients with pancreatic duct disruption recovered within 40 days favouring conservative approach (SEALANTS approach)
2.	Mohseni et al. [7]	Length of hospital stay was longer in the resected group. Morbidity and mortality were not significantly different
3.	Asensio et al. [8]	Higher mortality in patients undergoing pancreatoduodenectomy following trauma. (Including patients with complex grade five injuries)
4.	van der Wilden et al. [9]	Reviewed patients undergoing trauma Whipple’s procedure and concluded conservative procedure would be appropriate
5.	Thomson et al. [10]	Mortality of 33% in patients undergoing staged Whipple’s procedure following trauma

Patients who had a delay in diagnosis suffered from higher complication rates of 40% vs 18% in early diagnosed patients [2]. Treatment of grade four injuries with concomitant traumatic pancreatitis is controversial. The majority of recommendations are in favour of external drainage, thus avoiding resection [11,12]. The most common complication following pancreatectomy was fistula formation with an incidence of 5-37%. Most of these cases heal spontaneously over a period of time [13]. Suture closure of the distal pancreatic stump instead of pancreatico-enteric anastomosis has also been advocated when the bowel and pancreas are edematous and grossly inflamed [14]. Intraabdominal abscess formation is another common complication occurring in 10-25% of patients [13,15]. Early onset endocrine and exocrine deficiency following proximal or distal pancreatectomy for trauma are rare [16]. Reports of auto-islet transplant being attempted in order to achieve near-normal glucose tolerance following trauma Whipple's procedure has been successful [17,18].

Our patient had complete transection at the neck of the pancreas with the development of pancreatic ascites and acute pancreatitis. In this scenario, the endoscopic approach to treat duct disruption would have been futile. The stable hemodynamic status and absence of other organ injuries pushed us to proceed with the resection procedure. However, we were well aware of the risk factors involved, i.e., soft pancreas, pancreatic duct size less than one millimeter, indication for surgery other than adenocarcinoma [19], and acute pancreatitis, which form high-risk factors for pancreatic leak. Continuous irrigation has been successful in reducing complications following pancreatic surgeries [20]. We, therefore, irrigated the pancreatic anastomosis continuously with saline at 50ml/hour from day one. Could this be the reason our patient developed only biochemical leak? Further studies are required to prove or disprove the same. In the presence of the required expertise, we suggest that proceeding with resection in grade four pancreatic injuries should be considered as the first option in carefully selected patients. Morbidity following surgery is either comparable to or less than that occurring following non-resectional procedures. Prospective studies comparing resectional and non-resectional procedures are required to provide proper guidelines in the management of grade four pancreatic trauma.

## Conclusions

Pancreaticoduodenectomy for high grade pancreatic injuries still has a definite role in management under consideration of the possible complications with taking all the required precautions for early detection and treatment of possible complications. In selected patients, this can be the least morbid procedure providing a faster recovery. However, the surgery should be undertaken by hepatobiliary surgeons with experience in this area and at centers capable of handling such patients.
